# Retrobulbar Filling for Enophthalmos Treatment in Dogs: Technique, Description and Computed-Tomographic Evaluation. Preliminary Cadaveric Study

**DOI:** 10.3390/vetsci10040267

**Published:** 2023-03-30

**Authors:** Dario Costanza, Leonardo Meomartino, Barbara Lamagna, Erica Castiello, Pierpaolo Coluccia, Giuseppe Piegari, Ilaria D’Aquino, Francesco Lamagna, Adelaide Greco

**Affiliations:** 1 Interdepartmental Center of Veterinary Radiology, University of Napoli “Federico II”, Via Federico Delpino 1, 80137 Napoli, Italy; 2 Department of Veterinary Medicine and Animal Production, University of Napoli “Federico II”, Via Federico Delpino 1, 80137 Napoli, Italy; 3 Unit of Pathology, Department of Veterinary Medicine and Animal Production, University of Napoli “Federico II”, Via Federico Delpino 1, 80137 Napoli, Italy

**Keywords:** canine, entropion, eye, lipofilling, retrobulbar injection

## Abstract

**Simple Summary:**

In human medicine, a treatment modality for the sunken eye (enophthalmos) is retrobulbar fat injection. This preliminary study aimed to assess the feasibility and standardize the modalities of retrobulbar injection for enophthalmos treatment in dogs. Using an ultrasound-guided approach, two solutions, similar to adipose tissue, were injected within the retrobulbar space of dogs’ cadavers. The amount of solution to be injected was estimated using formulas described for ocular-regional anesthesia. Eyeball displacement was evaluated using two different computed tomography (CT)-based methods. The damage to retrobulbar structures due to the maneuver was assessed by necropsy and histopathological examination. A few milliliters of solution were needed to achieve the resolution of enophthalmos. The postmortem and histopathological examination did not find damage to the retrobulbar structures. Both proposed CT methods could quantify the eyeball displacement. Retrobulbar fat injection may represent a treatment modality for enophthalmos in dogs.

**Abstract:**

A new therapeutic approach for enophthalmos may be retrobulbar lipofilling. This study aims to standardize the intraconal filling technique and to evaluate the degree of eyeball displacement by computed tomography (CT). Skull CT was performed on six dog cadavers before and after intraconal injection of two 5% iodinated, viscoelastic solutions, one per eye, using an ultrasound-guided supratemporal approach. The volume to be injected was calculated using formulas for retrobulbar cone anesthesia. After CT, the dogs underwent necropsy and histopathology to evaluate damages that eventually occurred to retrobulbar structures. Eyeball displacement was estimated using two CT-based methods, named M_1_ and M_2_. The Wilcoxon signed-rank test revealed no significant difference between the two injected materials in both M_1_ (*p* > 0.99), and M_2_ (lateral *p* = 0.84 and rostral *p* = 0.84 displacement). A statistically significant difference was found between the pre- and post-injection group M_1_ (*p* = 0.002), M_2_ (*p* = 0.004) for the lateral and (*p* = 0.003) for rostral displacement. Although the slight eyeball displacement, the retrobulbar filling can lead to enophthalmos resolution. Compared to M_1_, the M_2_ method has better-defined anatomical landmarks. Further, preclinical in vivo studies are necessary to assess retrobulbar filling efficacy and safety.

## 1. Introduction

Enophthalmos is relatively common in dogs. It can result from Horner’s syndrome, fibrosis and/or atrophy of the orbital and masticatory muscles or fat due to myositis or cellulitis, orbital, nasolacrimal duct or retrobulbar neoplasia, tetanus, orbital fractures secondary to trauma or resulting from orbitotomy [1,2]. Severe enophthalmos can result in secondary entropion and prolapse of the nictitans membrane, occlusion of the visual axis and consequently, partial vision loss [3]. A solution may be the total resection of the third eyelid, but keratoconjunctivitis sicca, keratitis and corneal ulcers may result [3]. Furthermore, many dog breeds (i.e., Afghan Hound, Golden Retriever, Great Dane, Labrador Retriever, Newfoundland, Standard Poodle, Rottweiler and Weimaraner) could have enophthalmos due to the selective breeding processes for refined skull conformation [4]. The posterior eyeball displacement creates a pocket in the ventral conjunctival fornix, where foreign material accumulates, and an inward turning of the eyelid (entropion), leading to altered tear drainage and contact with the hairy eyelid skin to the ocular surface. This process results in chronic conjunctivitis, nictitans hyperemia, slight mucoid discharge (medial canthal pocket syndrome), irritation or pain and, potentially, corneal damage [4,5]. In the large and giant breeds of dogs with entropion and enophthalmos, entropion repair is less predictable due to the lack of contact between the globe and lower eyelid [6].

In human medicine, autologous fat intraconal injection (retrobulbar lipofilling) for enophthalmos resolution, mainly resulting from blow-out fractures or tumor resection, is well-described with excellent results and a low complication rate [7,8,9,10,11,12,13]. Among diagnostic imaging modalities, magnetic resonance imaging could provide detailed information regarding the nature and extent of concurrent soft tissue injuries within the retrobulbar space [14,15]. On the other hand, computed tomography (CT) is the modality of choice for evaluating the orbital bony structures [16,17,18,19]. Furthermore, CT, due to its wide distribution, speed of acquisition, high spatial resolution, multiplanar reconstructions (MPR), three-dimensional (3D) volume rendering, and the simultaneous visualization of the eyeball, skeletal structures, retrobulbar space and adnexa, is widely used for the evaluation of retrobulbar space [19,20,21,22,23,24].

In Veterinary Medicine, except for a single experimental study in rabbits [25], there are no published studies assessing the feasibility of intraconal lipofilling for enophthalmos resolution in dogs. In the Authors opinion, as in humans, intraconal lipofilling could be a valuable method for enophthalmos treatment. 

Therefore, the primary aims of this study were to describe and evaluate a technique to inject, within the retrobulbar space, two different materials, one per eye, with viscoelastic proprieties similar to the adipose tissue, and to evaluate the eyeball rostral displacement using CT. A secondary objective was to evaluate the presence of eventually occurring damages to retrobulbar structures through macroscopic and histopathological examination.

## 2. Materials and Methods

### 2.1. Study Protocol, Selection and Description of Subjects

The single-center experimental, prospective, one group pre-test, post-test, method comparison study was approved by the Clinical Ethical Review Board of the University of Napoli “Federico II” (PG/2022/0063335), and written owner consent was obtained for all the dogs included in the study. Dogs were included in the sample if: (a) were euthanized for reasons unrelated to this study, (b) had a certain degree of enophthalmos, (c) intraconal injection and necropsy were performed on the same day. Exclusion criteria were the presence of alterations found on clinical examination or following CT examination in the eyeballs, retrobulbar space and ocular adnexa. The skull morphotype (dolichocephalic, mesaticephalic, brachycephalic), sex, weight and age of each dog cadaver included in the final sample were recorded. Skull CT was performed on each dog cadaver using a 16-slice computed tomography unit (GE BrightSpeed, GE Healthcare, Milwaukee, WI, USA). The images were acquired using a standardized institutional protocol for the skull with the following parameters: sternal recumbency, tube potential 120 kVp, mA 180–250, rotation speed 0.8 s, slice thickness 0.625 mm, slice interval 0.625 mm, pitch: 0.925. Images were reconstructed using soft tissue and bone convolution algorithm (General Electric proprietary ‘standard’ and ‘bone’, respectively) with matching soft tissue window level (WL): 40, windows width (WW): 350 and bone windows (WL: 300, WW: 1500).

### 2.2. Retrobulbar Injections

**Figure 1:**
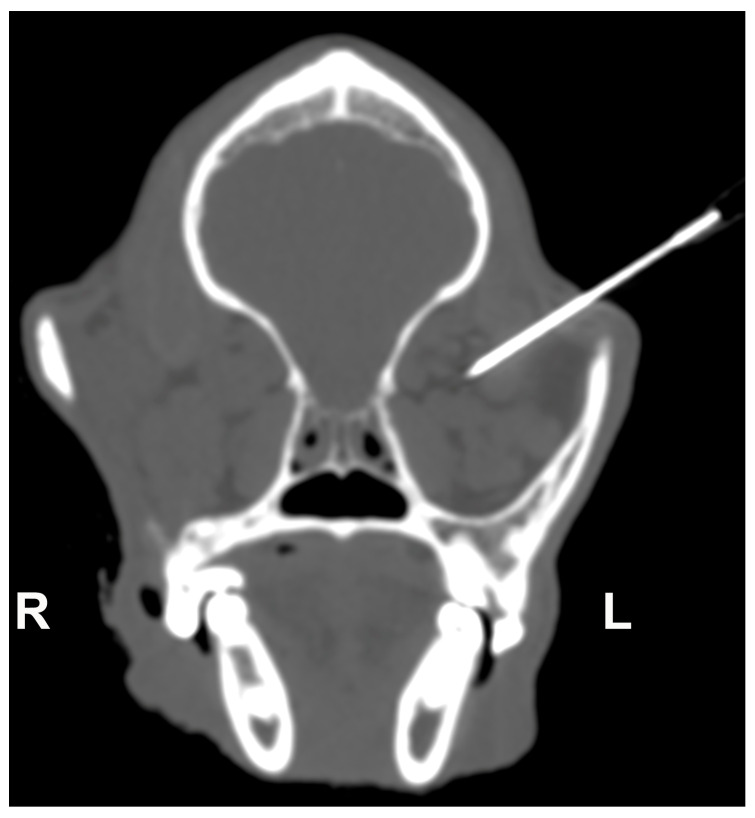
Transverse-oblique CT image of the skull obtained after needle insertion to assess the correct positioning within the left retrobulbar cone. R = right; L = left.

After CT acquisition, two viscoelastic solutions: (a) 3% sodium hyaluronate (VISCO-3 Sodium Hyaluronate, Zimmer Biomet, Warsaw, Poland) and (b) carmellose sodium gel with 2.5% lidocaine (Luan 2.5% gel, Molteni Farmaceutici S.p.A, Scandicci FI, Italy) were mixed with 1% methylene blue (Methylene Blue solution, Merck KGaA, Darmstadt, Germany) and 5% iodine-based, nonionic contrast medium (Iopamidol, Iopamiro 370 mgI/mL, Bracco Imaging S.p.A, Milano, Italy). Due to their composition, these solutions had both a viscosity component (the ability to deform under stress and maintain that deformation over time) and an elasticity component (the ability to return to its original volume after the stress is removed) and were consequently deemed similar to adipose tissue. The total volume of solution to be injected, expressed in mL, was calculated using the formulas proposed by Greco et al. [26] for retrobulbar cone volume, taking into account the skull morphotype and the weight of each dog. The solutions were injected within the retrobulbar space with an 18 Gauge needle using an ultrasound-guided supratemporal approach (Figure 1) [27]. The same operator executed all injections. Solution A was injected within the right retrobulbar space of each specimen, and solution B in the left one. After the injection, the CT exam was repeated using the same settings as the first acquisition.

### 2.3. Macroscopic and Histological Examination

After CT, a post-mortem examination was performed on all dog cadavers. Necropsies were performed in the necropsy room of the Department of Veterinary Medicine and Animal Production of the University of Napoli “Federico II” with a previously described standard necropsy protocol [28]. The eyeballs and retrobulbar spaces were evaluated to investigate the damage due to the retrobulbar filling maneuver. Eyeballs were also collected for histopathologic examination; samples were fixed in 10% neutral buffered formalin, embedded in paraffin, sectioned at 4 microns and stained with hematoxylin and eosin (HE) for morphological evaluation of lesions [29].

### 2.4. CT Image Analysis

**Figure 2:**
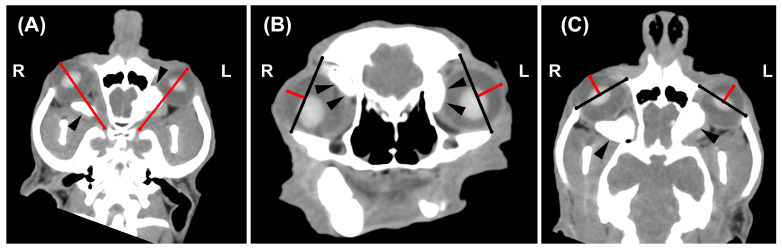
Dorsal-oblique (**A**), transverse (**B**) and dorsal (**C**) post-injection MPR image of the skull of a 17-year-old male mixed-breed dog. Soft tissue algorithm (manually windowed to WW = 350, WL = 40), slice thickness 0.625 mm, kVp 120, mAs 220). (**A**) In M_1_, the eyeball displacement was evaluated on a dorsal-oblique plane, drawing a line from the optic foramen to the ipsilateral corneal surface (red lines). (**B**) In M_2_, the lateral displacement was assessed on the transverse plane, drawing a line from the frontal to the zygomatic bone (black lines) and then from this line to the corneal surface (red lines), while the rostral displacement was evaluated on the dorsal plane (**C**), drawing a line from the maxillary to the zygomatic bone (black lines) and then from this line to the corneal surface (red lines). The contrast media mixed with the viscoelastic solutions (black arrowheads) is visible within the retrobulbar space. R = right; L = left.

Pre- and post-injection CT images of each dog were compared by a professor of Veterinary Radiology with a Ph.D. and >25 of experience (L.M.) on a computer workstation (iMac 5K, 27-inch, Apple Inc., Cupertino, CA, USA) using an open-source DICOM viewer (Horos version 3.3.6, 64-bit, Nimble Co LLC d/b/a Purview, Annapolis, MD, USA, https://www.horosproject.org, accessed on 20 November 2022). Any eventual intraocular or extraconal distribution of the contrast medium was recorded. Eyeball displacement was estimated subjectively, observing the macroscopic position of the eyeballs, and objectively, using two methods, named M_1_ and M_2_. In M_1_ (Figure 2A), the rostro-lateral displacement was evaluated on a dorsal-oblique plane tracing a line from the corneal surface to the optic canal. In M_2_, the lateral displacement was assessed on the transverse plane (Figure 2B), drawing a line from the frontal to the zygomatic bone and then from this line to the corneal surface, while the rostral displacement was evaluated on the dorsal plane (Figure 2C), drawing a line from the maxillary to the zygomatic bone and then from this line to the corneal surface.

### 2.5. Statistical Analyses

Statistical analyses were performed by one of the authors (D.C.), a third-year Ph.D. student in Veterinary Diagnostic Imaging, using commercial statistical software (Prism version 9.5.0 (525), GraphPad Software San Diego, CA, USA). Descriptive statistics were calculated for both the pre- and post-injections groups using M_1_ and M_2_. The Wilcoxon signed-rank test was used to assess the presence of significant differences between the left and right eyeballs in pre- and post-injection groups and then to evaluate the degree of rostro-lateral displacement for both methods. In all analyses, *p* < 0.05 was considered statistically significant.

## 3. Results

### 3.1. Population

The final sample consisted of six dogs [three females (one spayed) and three intact males) (four mesaticephalic and two brachycephalic)] for a total of 12 retrobulbar spaces. The breed represented were mixed breed (*n* = 4), English Cocker spaniel (*n* = 1), and Chihuahua (*n* = 1). The median weight was 9.4 kg (range 5–12.7 kg) and median age was 6 years (range 3–17 years).

### 3.2. Retrobulbar Injections Findings

**Figure 3:**
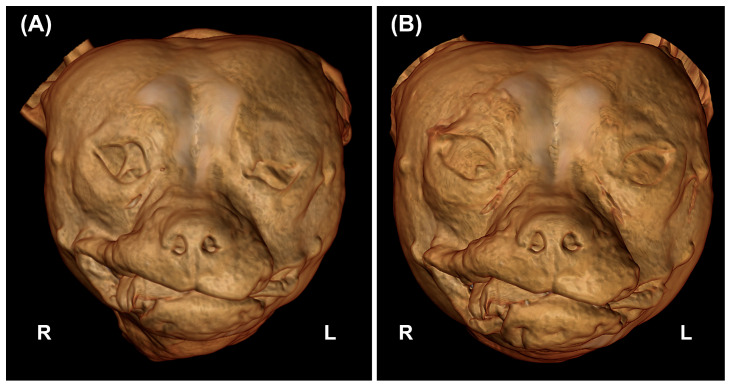
Three-dimensional (3D) volume rendering images of the same dog. Pre- (**A**) and post-injection (**B**). In (**B**), there is a slight but visible rostral advancement of both eyeballs compared to image (**A**), highlighted by the more opened eyelids. R = right; L = left.

The volume injected was between 1.1–1.6 mL per eye. The ultrasound-guided intraconal filling was achieved in all the dogs without significant difficulties. An evident rostral displacement of the eyeballs was subjectively deemed adequate for all the dogs (Figure 3).

### 3.3. Necropsy Macroscopic Examination and Histological Findings

**Figure 4:**
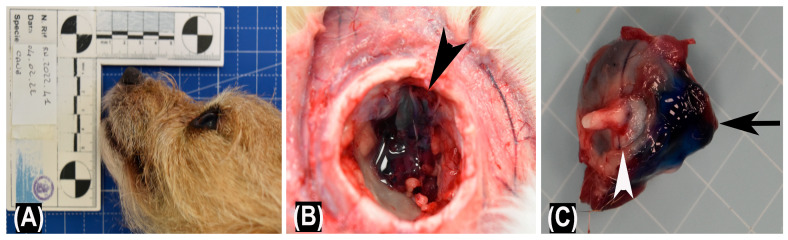
Representative macroscopic findings in treated cadavers. (**A**) Close-up view of dog cadaver head with normal positioning of the eyeball. (**B**) The injected solution is visible within the retrobulbar space (arrowhead). (**C**) The injected solution is visible on the caudal surface of the eyeball (black arrow) without signs of post-mortem damage to the eyeball or optical nerve (white arrowhead).

The macroscopic examination showed a rostral displacement of the treated eyeballs in all assessed cadavers (Figure 4A). The eyeballs and the optic nerve showed no signs of post-mortem tissue damage. Furthermore, following eyeballs enucleation, the injected material was observed both within the retrobulbar space (Figure 4B) and on the caudal surface of the eyeballs (Figure 4C).

**Figure 5:**
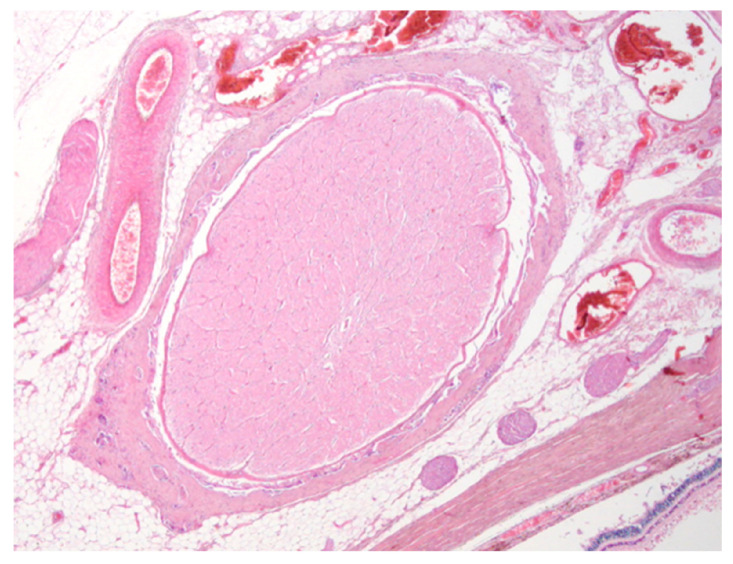
Representative histologic cross section of the optic nerve (hematoxylin and eosin stain, original magnification 2×).

The histopathological examination showed no signs of post-mortem tissue damage involving ocular structures in all the cases (Figure 5).

### 3.4. CT Images Findings

The contrast medium, mixed within the injected solutions, was visible within all the retrobulbar spaces. The iodinated viscoelastic solution was completely intraconal in seven retrobulbar spaces, while it was partially or completely in the extraconal space in three and two retrobulbar spaces, respectively.

### 3.5. Statistical Results

Descriptive statistics, including the mean, range (minimum to maximum), standard deviation (±SD) and 95% confidence interval (CI) of the mean, for eyeball displacement calculated for the left, right and pooled eyeballs in both pre- and post-injection groups considering both M_1_ and M_2_ are reported in Table 1 and Table 2, respectively.

**Figure 6:**
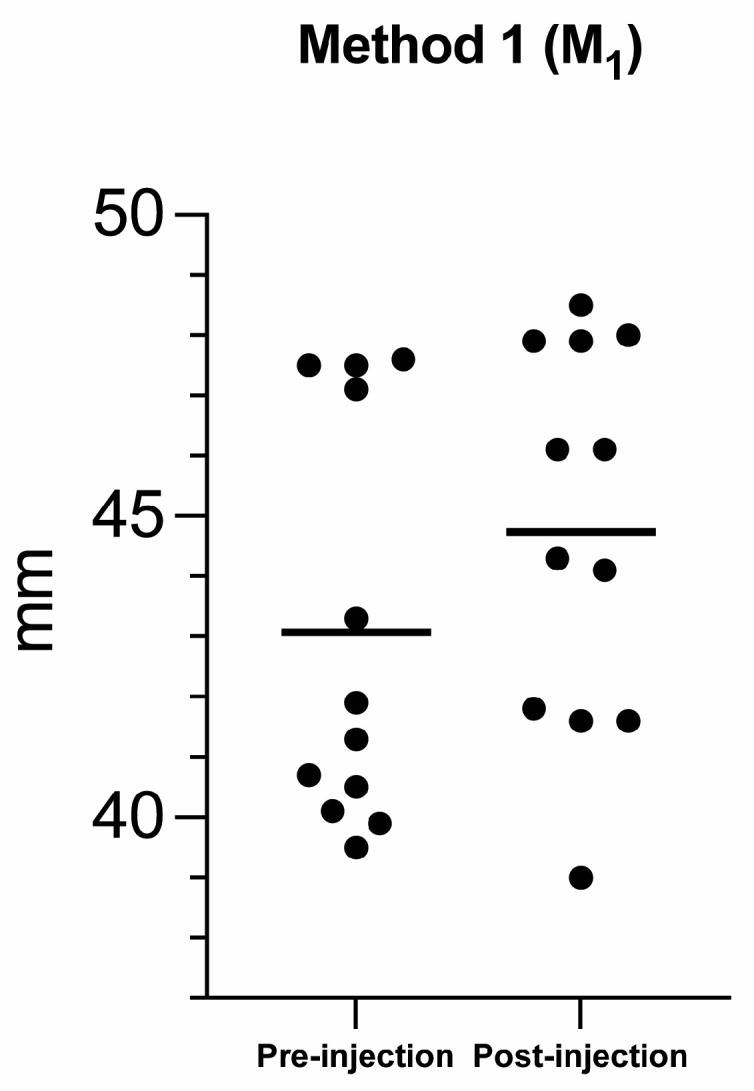
Scatter plot comparing the rostro-lateral displacement in millimeters (mm) between the pre- and post-injection groups using Method 1 (M_1_). The solid black lines represent the mean.

**Figure 7:**
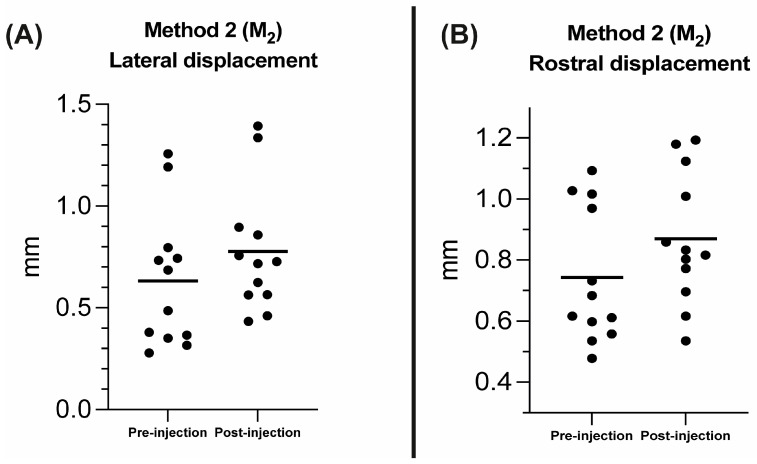
Scatter plots comparing the lateral (**A**) and rostral (**B**) displacement in millimeters (mm) between the pre- and post-injection groups using Method 2 (M_2_). The black solid lines represent the mean.

The Wilcoxon signed-rank test revealed no significant difference between the right and left eyeballs in the pre-injection group for the rostro-lateral displacement in M_1_ (*p* = 0.53), and for the lateral (*p* = 0.31) and rostral (*p* > 0.9) displacement in M_2_. No significant differences were also found between the right and left eyeball in the post-injection group for the rostro-lateral displacement in M_1_ (*p* > 0.99), and for the lateral (*p* = 0.84) and rostral (*p* = 0.84) displacement in M_2_. Consequently, the eyeballs and respective retrobulbar spaces were pooled for the following statistical analyses. The Wilcoxon signed-rank test revealed a statistically significant difference between the pre- and post-injection group for the pooled data for the rostro-lateral displacement in M_1_ (*p* = 0.002; mean percentage (%) = 5%; Figure 6), lateral (*p* = 0.004; mean percentage (%) = 35.2% Figure 7A) and rostral displacement in M_2_ (*p* = 0.003; mean percentage (%) = 19.3% Figure 7B).

## 4. Discussion

The primary aims of this study were to describe a technique to treat enophthalmos by injecting two different materials, with viscoelastic proprieties similar to the adipose tissue within the retrobulbar space and to evaluate the eyeball rostro-lateral displacement using CT. The results demonstrated that retrobulbar filling is a technique relatively simple to perform and without any contraindications. 

The injection technique adopted was derived from the supratemporal approach proposed by Chiavaccini et al. [27]. However, differently from the original method, the injection was performed under ultrasound guidance to avoid possible damage to the eyeballs and retrobulbar structures (i.e., the optic nerve and the vessels). The degree of eyeball rostral and lateral displacement was subjectively considered good, and despite using only a few mL ofmaterial, it was sufficient to obtain the de-sired result. 

Additionally, the eyeball rostral and lateral displacement was objectively evaluated using two different CT-based methods. The statistical analyses found no significant differences between the two injected materials. Additionally, both CT methods we proposed to evaluate the degree of rostral and lat-eral displacement of the eyeball were found to be reliable. These methods confirmed a significant displacement compared to the starting condition. However, although M_1_ allows the assessment of the rostral and lateral eyeball advancement with a single measurement, M_2_ provides more defined anatomical landmarks and makes it easier to compare the pre- and post-injection series. Our methods are similar to the lateral and superior protrusion indexes described by Ye et al. [7] in human medicine and confirm the usefulness of CT in pre-operative planning and evaluation of the degree of correction of enophthalmos.

In human medicine, only a few studies estimated the amount of fat to be injected before the retrobulbar filling [7,8]. In others, the amount of autologous fat to be injected was at the clinician’s discretion according to the aesthetic improvement achieved [9,11,12,13]. Its lack of precision may be responsible for early diplopia and unsatisfactory aesthetic results requiring additional injections [7]. In our study, the volume to be injected was estimated by formulae proposed by Greco et al. [26] to calculate the retrobulbar cone volume using CT. This method has proved to be effective; however, for in vivo inoculations, considering that a reabsorption rate of up to 30–40% of the injected autologous fat is expected in a few weeks, this loss must have to be taken into account; otherwise, additional inoculations could be necessary [7,8,9].

In our study, the injected iodinated viscoelastic solutions were partially or completely extraconal in three and two cases, respectively. This finding is most likely related to the author’s inexperience in performing this injection but probably also to a partial leakage of the more fluid component of the solution from the intraconal space. The inoculation of fat within the extraconal space can cause a slight swelling of the eyelids, inadequate cranial displacement of the eyeball and post-operative transitory pain. However, according to the literature, extraconal migration of autologous fat does not alter the function of extraocular muscles, optic vessels, and nerves [7,9,11,13]. In human medicine, hard materials such as hydroxyapatite, cartilage, silicone or glass beads have been implanted in the orbit for the same purpose. However, these materials do not meet the physiologic and anatomical requirements of the orbit, so major complications such as vision loss, ocular motility restriction, ocular nerve and vessel damage, and migration of these implants can occur [7,9,11,17]. Minor complications related to autologous fat inoculation and reported in the literature include intraoperative retrobulbar hemorrhage, post-operative pain, periorbital swelling, ptosis and blurring of the vision. Most of these complications tend to resolve in a short period and without irreversible damage to relevant anatomical structures [9,13]. Potential major complications such as acute blindness, fat embolism and oculo-cardiac reflex have not been reported so far [9,11,30]. The method’s safety was also confirmed in our study by the absence of relevant damages at the necropsy and histopathological exam. Indeed, no injury to the eyeball, the ocular muscles, vessels and optic nerve was demonstrated. Obviously, further preclinical studies on living subjects are necessary to assess the number and extent of complications following this procedure.

The main limitations of our study are represented by the small sample size and its cadaveric nature. Therefore, we were unable to clinically confirm the absence of damage to the optical structures and other major complications, such as vision loss. Additionally, another limitation is represented by the type of materials used, which although viscoelastic, has different physical properties compared to autologous fat, so the amount of material to be inoculated and the results obtained from our injection may differ in vivo.

## 5. Conclusions

The retrobulbar filling could represent a possible technique for treating enophthalmos in dogs. The supratemporal approach adopted for the injection maneuver has proven to be relatively simple and safe, without macroscopic and histopathological lesions to the retrobulbar structures. Both proposed CT methods used to estimate the degree of ocular displacement were able to detect the degree of rostral and lateral displacement of the eyeball. Further, in vivo studies are necessary to investigate the procedure’s effectiveness, the modalities and sites of autologous fat harvesting and the rate of major complications.

## Figures and Tables

**Table 1:** Descriptive statistics for M_1_ pre- and post-retrobulbar injection.

	Mean (±SD)	95% CI	Range (Min–Max)
Pre-RT (n = 6)	42.93 (±3.44)	39.32–46.55	39.9–47.5
Pre-LE (n 0 6)	43.22 (±3.59)	39.44–46.99	39.5–47.6
Post-RT (n = 6)	45.03 (±2.97)	41.91–48.15	41.6–48.5
Post-LE (n = 6)	44.45 (±3.61)	40.66–48.24	39–48
Pre-Grouped (n = 12)	43.08 (±3.36)	40.94–45.21	39.5–47.6
Post-Grouped (n = 12)	44.74 (±3.16)	42.73–46.75	39–48.5

Abbreviations: CI, confidence interval; Grouped; grouped data of right and left eyeballs; Post, post-injection; Pre, pre-injection; LE, left; Min, minimum; Max, maximum; RT, right; SD, standard deviation.

**Table 2:** Descriptive statistics for M_2_ pre- and post-retrobulbar injection.

	Displacement	Mean (±SD)	95% CI	Range (Min–Max)
Pre-RT (*n* = 6)	LD	0.63 (±0.37)	0.24–1.02	0.27–1.25
	RD	0.72 (±0.24)	0.46–0.98	0.47–1.09
Pre-LE (*n* = 6)	LR	0.62 (±032)	0.28–0.96	0.31–1.19
	RD	0.76 (±0.21)	0.53–0.98	0.55–1.02
Post-RT (*n* = 6)	LD	0.78 (±0.33)	0.43–1.13	0.46–1.39
	RD	0.87 (±0.26)	0.60–1.15	0.53–1.19
Post-LE (*n* = 6)	LD	0.77 (±0.31)	0.44–1.1	0.43–1.33
	RD	0.85 (±0.18)	0.66–1.04	0.61–1.12
Pre-Grouped (*n* = 12)	LD	0.63 (±0.33)	0.42–0.84	0.27–1.25
	RD	0.74 (±0.22)	0.60–0.88	0.47–1.09
Post—Grouped (*n* = 12)	LD	0.77 (±0.30)	0.58–0.97	0.43–1.39
	RD	0.86 (±0.21)	0.73–1	0.53–1.19

Abbreviations: CI, confidence interval; Grouped; grouped data of right and left eyeballs; Post, post-injection; Pre, pre-injection; LE, left; LD, lateral displacement of the eyeball; Min, minimum; Max, maximum; RD, rostral displacement of the eyeball; RT, right; SD, standard deviation.

## Data Availability

The data supporting the findings of this study are available from the corresponding author, upon reasonable request.
